# Home Meal Replacement Fortified with Eggshell Powder and Vitamin D Prevents Bone Loss in Postmenopausal Women: A Randomized, Double-Blind, Controlled Study

**DOI:** 10.3390/nu16081152

**Published:** 2024-04-12

**Authors:** Nam-Seok Joo, So-Hui Shin, Kyu-Nam Kim, Seok-Hoon Lee, Susie Jung, Kyung-Jin Yeum

**Affiliations:** 1 Department of Family Practice and Community Health, Ajou University School of Medicine, Suwon 16499, Republic of Korea; jchcmc@hanmail.net (N.-S.J.); ktwonm@hanmail.net (K.-N.K.); dgwho@naver.com (S.-H.L.); lovehrh@naver.com (S.J.); 2 Department of Food and Nutrition, College of Biomedical and Health Science, Konkuk University, Chungju 27478, Republic of Korea; ssh9936@kku.ac.kr

**Keywords:** natural calcium, bone mineral density, parathyroid hormone

## Abstract

Calcium and vitamin D deficiencies have been ongoing problems in Koreans due to a lack of food sources of calcium and vitamin D. Postmenopausal women aged 50 to 64 years (*n* = 25) were randomly assigned to consume three home meal replacements (HMRs)/week with (treatment) and without (control) eggshell powder and vitamin D for 6 months. Additionally, subjects who agreed to continue the study consumed the same three HMRs/week for an additional 6 months in this randomized double-blind study. We confirmed the high compliance of the study participants by analyzing carotenoids, the bioactive substances of HMRs, in the blood. The treatment group consumed an additional 261 mg/d of calcium and 10.3 μg/d of vitamin D from the HMRs, thus meeting the recommended intakes of calcium and vitamin D for Koreans. As a result of consuming fortified HMRs for 6 months, the decline in femoral neck bone density was significantly reduced in the treatment group (*p* = 0.035). This study indicates that inexpensive eggshell powder may be a good source of calcium for populations with low consumption of milk and dairy products. Additionally, functional HMRs fortified with eggshell powder and vitamin D can be a good dietary strategy for bone health.

## 1. Introduction

Osteoporosis, one of the major health problems of postmenopausal women, increases fracture-related mortality [1]. Considering that the estrogen level drops during the menopausal transition period result in more bone resorption than formation, proactive measures are needed for postmenopausal women. There is no question that calcium and vitamin D are essential nutrients that play an important role in bone health [2,3,4,5]. However, despite general recommendations for adequate calcium and vitamin D intake for bone health, there is still controversy regarding the effectiveness of supplementation with these nutrients for bone health and fracture prevention [6,7].

Calcium intake in many Asian countries, including China [8], India [9], Japan [10,11], and Korea [12], has been reported to be very low, in the range of 300 to 600 mg/d, and are associated with a higher risk of fractures in older women [3,13]. The high incidence of lactose malabsorption in Asian countries can be a major obstacle to the consumption of milk and dairy products, which are the main source of dietary calcium [14]. In these Asian countries, the prevalence in the population of lifetime lactase-phlorizin hydrolase is surprisingly low, reported at 0% in South Korea, and 15% in China [15]. Therefore, there is an urgent need to find alternative sources of dietary calcium along with vitamin D for these populations.

Carotenoids, fat-soluble pigments that give colors such as yellow, orange, and red, are abundant in brightly colored fruits and vegetables [16]. Green vegetables such as spinach are rich in xanthophylls such as lutein, orange vegetables such as carrots are rich in β -carotene, and red vegetables and fruits such as tomatoes and watermelon are rich in lycopene [17]. These carotenoids are well known to be bioavailable and to have biological functions, including antioxidant, anti-inflammatory, anticancer, and reducing the risk of cardiovascular disease and macular degeneration [18]. Recently, the effectiveness of lycopene in preventing bone loss has been reported in animal models [19]. 

An interesting attempt has been proposed to increase calcium and reduce lactose concentration by treating milk with eggshells and kefir grains [20]. Eggshells are mainly composed of calcium carbonate [21] and have been reported to contain approximately 39% calcium [22]. Additionally, a vitamin–mineral supplement enriched with chicken eggshell powder has been reported to increase femoral neck mineral density in postmenopausal women consuming more than 1200 mg of calcium per day [23]. Daily consumption of foods high in natural calcium, such as eggshells, may be a better dietary strategy to ensure sustainable calcium intake than taking supplements. It is noteworthy that the home meal replacement (HMR) industry is growing rapidly due to the increase in the elderly population and single-person households worldwide [24]. Additionally, reflecting the desire for a healthy life, the demand for functional, customized HMRs is being emphasized more than ever [25]. 

Therefore, a randomized, double-blind, controlled intervention study was conducted to determine the effect of eggshell and vitamin D-fortified HMRs on bone mineral density in postmenopausal women with insufficient calcium intake.

## 2. Materials and Methods

### 2.1. Study Design

**Figure 1:**
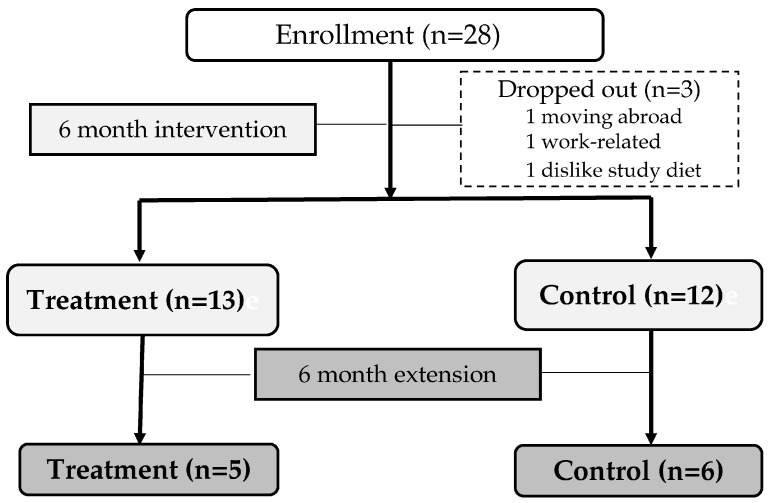
Study participant enrollment and study flow.

This study is a randomized, double-blind, controlled study conducted in postmenopausal women. Study participants were randomly assigned (stratification variables; age and body mass index) to consume either (1) three home meal replacements (HMRs) per week consisting of curry, black bean sauce and sweet pumpkin porridge (control), or (2) three home meal replacements consisting of curry, black bean sauce, and sweet pumpkin porridge, fortified with eggshell powders and vitamin D (treatment) for 6 months. Subjects enrolled in this study were randomly assigned 1:1 to the intervention and control groups, and the randomization code was managed by an independent person in charge of the clinical trial center. Double-blindness was maintained for the investigators and subjects. The taste of the HMRs consumed by the treatment and control groups was similar, and the packaging of the HMRs was the same between the two groups. There was no difference in the composition of black bean paste and sweet pumpkin porridge between the control and treatment groups except for eggshell powder and vitamin D, but in the case of curry, tomatoes were added along with eggshell powder and vitamin D only in the treatment group. To determine study subjects’ compliance with the dietary intervention and the accuracy of the assigned diet intake, the carotenoid content of the study diet and serum carotenoid concentration were analyzed. It was expected that the blood concentration of lutein, which is abundant in sweet pumpkin porridge, would increase in both the control and treatment groups. On the other hand, considering that tomatoes were added to the curry only in the treatment group, serum lycopene concentration was expected to increase only in the treatment group. Study participants who agreed to extend the intervention period for an additional 6 months were provided with the same HMR as for the first 6 months. Except for the study diet, study subjects were instructed to consume their daily diet and not to change their usual exercise levels during the intervention period. The study flow and number of study participants are shown in Figure 1. Study participants consumed the remaining meals as usual except for the three HMRs provided each week. The study protocol was approved by the Institutional Review Board of Ajou University Hospital and written informed consent was obtained from all study participants.

### 2.2. Participants 

Twenty-eight participants were enrolled in this study, and three participants withdrew due to personal reasons such as moving abroad, work-related issues, or disliking the study diet. Study participants who were postmenopausal women between the ages of 50 and 64 (12 months of spontaneous amenorrhea, if the last menstrual period was less than 6 months to 1 year and blood follicular stimulating hormone (FSH) > 40 mIU/mL) with a body mass index of 18.5 to 30.0 kg/m^2^ were enrolled in this study. Subjects who had a T-score < −2.5 (the L1 to L5 lumbar spine average, femoral neck or total hip); had received calcium supplements, biophosphonate treatment, vitamin D supplements exceeding 200 IU per day, and hormone replacement therapy within 3 months prior to screening; whose aspartate aminotransferase (AST) or alanine aminotransferase (ALT) exceeded 120 U/L; whose creatinine exceeded 2.0 mg/dL; who were suspected of having abnormal thyroid function; who had thyroid stimulating hormone (TSH) levels outside the normal range, thyroid cancer or hyperfunction; or uncontrolled hypertension (>106/100 mmHg); or had uncontrolled diabetes (fasting blood sugar over 180 mg/dL, new medication added due to diabetes within 3 months); a history of malignancy; an esophageal disease that delays esophageal emptying; uncorrected hypercalcemia/hypocalcemia; uncontrolled chronic disease that can affect metabolism (chronic, liver diseases, alcoholism, primary hyperparathyroidism); drug treatment that affects bone or calcium metabolism (steroids, diuretics, etc.); or clinically significant diseases and egg allergy, were excluded from the study. Twenty-five subjects (13 in the treatment group, 12 in the control group) successfully completed the 6-month intervention, and 11 subjects (5 in the treatment group, 6 in the control group) who agreed to an extension for an additional 6 months completed the 12-month intervention.

### 2.3. Anthropometry, Biochemical Analysis, Bone Mineral Density Measurement, and Assessment of Dietary Intake

Body mass index, waist circumference, and blood pressure were measured at each visit (baseline, 3, 6, 9, and 12 months). Lipid profile, glucose, hemoglobin A1c, insulin, calcium, osteocalcin, parathyroid hormone (PTH) and 25-hydroxyvitamin D [25(OH)D] were determined in eight-hour fasting serum. Additionally, urinary n-telopeptide, calcium, and creatinine were also measured in spot urine. Serum 25(OH)D concentrations were measured with a radioimmunoassay kit (DiaSorin Inc., Stillwater, MN, USA) using a γ-counter (1470Wizard; PerkinElmer, Turku, Finland). Serum intact PTH was analyzed using a chemiluminescence assay (DiaSorin, USA). The bone mineral density (BMD) was measured in the lumbar spine (L1–4), total hip, and femoral neck by dual-energy X-ray absorptiometry (DXA, DISCOVERY-W fan-beam densitometer, Hologic Inc., Marlborough, MA, USA) with CVs of 1.9% and 2.5%, respectively. Dietary intake was analyzed through 3-day dietary records (two times on weekdays, one time on weekends) and a food frequency questionnaire using a computer-aided nutritional analysis program (Can Pro, web ver.5.0, The Korean Nutrition Society, Seoul, Republic of Korea).

### 2.4. Dietary Carotenoid and Serum Carotenoid Analysis

β-carotene (type IV), α-carotene, lycopene and lutein standards were purchased from Sigma-Aldrich Chemical Co (St Louis, MO, USA) and Kemin Industries (Des Moines, IA, USA), respectively. All UPLC solvents and water were purchased from Honeywell Burdick & Jackson (Muskegon, MI, USA) and filtered through a 0.45 μm filter before use. 

Extraction of carotenoids from serum and study diets was performed using the slightly modified Folch method [26] and tetrahyrofuran as described previously [27] with minor modification, respectively, and the analysis of carotenoids was conducted using a UPLC system (ACQUITY UPLC I-Class, Waters Co., Milford, MA, USA) equipped with a BEH C18 column (1.7 μm, 2.1 × 50 mm, Waters Co., Milford, MA, USA), C18 guard column, binary pump delivery system, autosampler, and photodiode array detector. The mobile phase consisted of acetonitrile/methanol (70:30, *v*/*v*) for solvent A, and water for solvent B. The flow rate was 0.5 mL/min, and the gradient was 78% solvent A and 22% solvent B held for 0.5 min, followed by linear gradient (6 min) to 94% solvent A and 6% solvent B, followed by linear gradient (1.0 min) to 100% solvent A and held for 5.5 min, then returned to initial condition and held for 2.5 min for equilibration. Carotenoids (at 450 nm) were quantified with each external standard curve, and each peak was confirmed by retention time and unique spectrum. The inter-assay coefficient of variation (CV) was under 4% (*n* = 10) and the intra-assay CV was under 4% (*n* = 10).

### 2.5. Lifestyle Habits Evaluation

Current smokers were defined as those who were currently smoking and had smoked more than five packs of cigarettes during their whole life. All others were regarded as ex-smokers or non-smokers. Regular alcohol drinkers were defined as those who drank alcohol more than once per month. All others were regarded as non-drinkers. Aerobic exercise was defined as more than 2 h and 30 min of moderate-intensity physical activity per week, more than 1 h and 15 min of high-intensity physical activity per week, or a combination of moderate-intensity and high-intensity physical activity. Lifestyle questions were asked to obtain basic information at the hospital, and responses were recorded after being explained by a research nurse through face-to-face interviews with the research subjects.

### 2.6. Statistical Analysis

All continuous variables, such as age, body proportions, metabolic parameters (glucose, cholesterol), hormones, bone markers, and bone mineral density, were compared by non-parametric comparison using the Mann–Whitney U test in two groups due to the small sample size. In case of categorical variables, such as smoking, alcohol drinking, and regular exercise, the non-parametric X^2^ test was used. Some variable changes, such as PTH, osteocalcin, NTx, and 25(OH)D, were compared by non-parametric Spearman correlation. Values are presented as mean ± standard deviation in tables and standard error in figures. The *p*-value < 0.05 was considered statistically significant. Statistical analyses were performed using SPSS (version 25.0; SPSS Inc., Armonk, NY, USA). 

## 3. Results

### 3.1. Similar Baseline Characteristics of Control and Treatment Groups

There were no significant differences between the control and treatment groups at baseline in general characteristics such as age, weight, body mass index, and waist circumference as shown in Table 1. Additionally, no significant differences were found in clinical characteristics except cholesterol, which was significantly higher in the treatment group (236 mg/dL) compared to the control group (190.5 mg/dL). Bone turnover markers such as serum osteocalcin and urine *N*-terminal telopeptides of type I collagen (NTx), and serum vitamin D, calcium, and parathyroid hormone levels, were similar between the two groups. In addition, there was no significant difference in bone mineral density of the lumbar spine, femoral neck, and total hip between the two groups. There were also no significant differences in energy, macronutrient, calcium, and vitamin D intake between the two groups at baseline. Although there was no significant difference in exercise time, there was a significant difference in the number of subjects who answered ‘yes’ to the question about regular exercise (control group vs. treatment group: 83.3% vs. 92.3%) at baseline.

### 3.2. Calcium, Vitamin D, and Carotenoid Contents in Study Diets

The study diet contained an average of 609.4 mg/serv. of calcium and 24.1 μg/serv. of vitamin D for the treatment group, and had only 129.2 mg/serv. of calcium for the control group as presented in Table 2. Since study participants consumed the study diets three times per week, they consumed 261 mg/d of calcium and 10.32 μg/d of vitamin D in the treatment group and 55.37 mg/d of calcium in the control group from the study diet during the intervention period. In addition, only the curry in the treatment group contained 6.608 mg/serv. of lycopene, whereas the amounts of lutein and β-carotene in both the control and treatment groups were similar. As a result, during the intervention period, the treatment group consumed an average of 0.944 mg lycopene, 0.973 mg lutein, and 0.887 mg β-carotene per day, and the control group had an average of 1.001 mg lutein and 0.931 mg β-carotene per day from the clinical diet.

### 3.3. Calcium and Vitamin D Intake of Study Participants 

**Figure 2:**
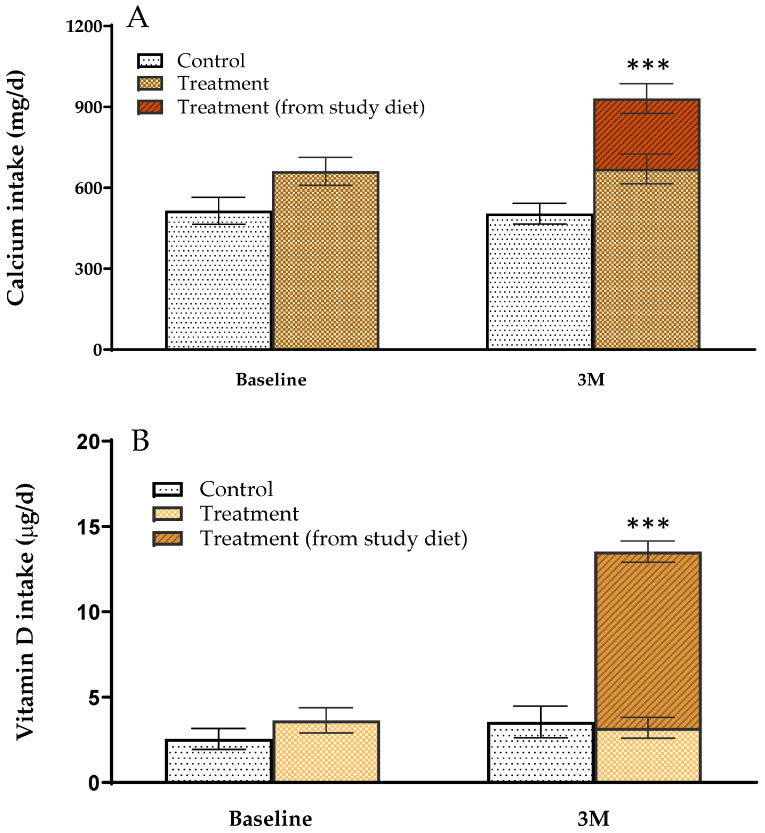
Calcium (**A**) and vitamin D (**B**) intake of study participants during the intervention period. Values are mean ± standard error and significant differences between groups were compared by non-parametric comparison using Mann–Whitney U test, *** *p* < 0.001. Calcium and vitamin D intake were determined by 3-day dietary records (two times on weekdays, one time on weekends). Study diets were analyzed by ICP-MS for calcium and LC-MS/MS for vitamin D.

There was no significant difference in calcium intake between the control (515 mg/d) and the treatment (661 mg/d) groups before the start of the intervention, with both groups having inadequate intake (Figure 2A). Additionally, vitamin D intake in the control (2.55 μg/d) and treatment (3.65 μg/d) groups was also insufficient at baseline (Figure 2B). However, study participants in the treatment group met the recommended intake of calcium (Figure 2A) and adequate intake of vitamin D (Figure 2B) for Koreans by consuming the study diet three times a week during intervention period. 

### 3.4. Changes in Serum Carotenoid Concentrations of Study Participants 

**Figure 3:**
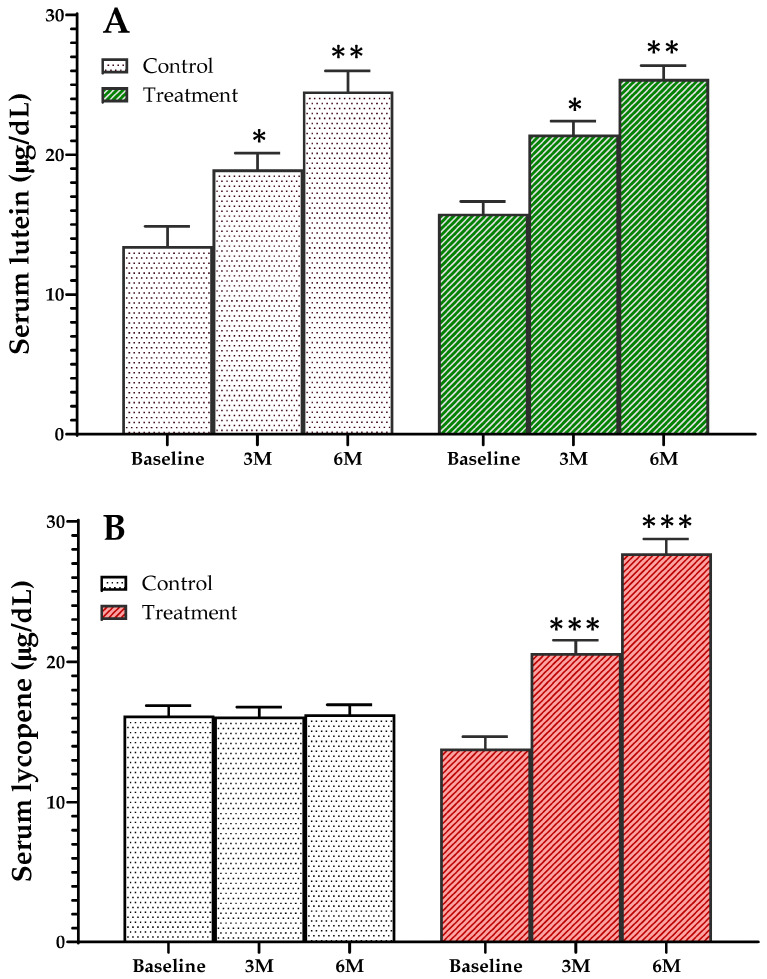
Changes in serum lutein (**A**) and lycopene (**B**) concentrations of study participants during the intervention period. Values are mean ± standard error and significant differences from baseline were compared by non-parametric comparison using Mann–Whitney U test, * *p* < 0.05, ** *p* < 0.01, *** *p* < 0.001.

Plasma lutein concentrations of study participants in both control and treatment groups significantly increased at 3 months (*p* < 0.05) and at 6 months (*p* < 0.01) after consumption of the study diet (Figure 3A). However, lycopene concentrations were significantly increased in the treatment group only at 3 months (*p* < 0.01) and at 6 months (*p* < 0.001), whereas the control group remained the same (Figure 3B).

### 3.5. Changes in Bone Mineral Density and Bone Markers of Study Participants

As a result of consuming the study diet fortified with natural calcium and vitamin D for six months, the decrease in bone mineral density of the femoral neck in the treatment group was significantly smaller than that in the control group (*p* = 0.035) as shown in Table 3. There was no significant difference in other bone markers between the two groups. 

Even though a small number of subjects (*n* = 11) successfully completed the 12-month intervention, changes in serum carotenoid concentrations were similar to results at 6 months. In addition, although there was no significant difference between the two groups (*p* = 0.052), the decrease in bone mineral density of the femoral neck in the treatment group (−0.007, *n* = 5) was smaller than that in the control group (−0.033, *n* = 6) as presented in Table 4. 

## 4. Discussion

In this randomized, double-blind, controlled intervention study, we found that consuming a home meal replacement (HMR) fortified with natural calcium and vitamin D can prevent rapid bone loss in postmenopausal women. Our finding that the chicken eggshell natural calcium can efficiently prevent bone loss is consistent with a previous study reporting the beneficial effects of a vitamin–mineral supplement enriched with chicken eggshell powder [23]. However, we believe this study is the first successful attempt to prevent bone loss in a population with insufficient calcium and vitamin D intake through a regular diet fortified with chicken eggshell powder and vitamin D rather than supplements. 

Although adequate calcium intake is important for overall health [28], insufficient dietary calcium intake is widespread worldwide [29], with Asians, Africans, and South Americans specifically failing to meet recommended levels [30]. In particular, in a recent study, the incidence of osteoporosis among postmenopausal women was reported to be 38.4% in Korea [31] and 30.3% in China [32]. In an environment where calcium and vitamin D intake is insufficient, an important issue is how to increase calcium and vitamin D intake through daily diet. It is reasonable to consider developing appropriate strategies to increase calcium intake in these populations for bone health. In general, it is recommended to consume dairy products regularly in daily life [33,34] or to consume calcium-fortified foods for bone health [35,36]. Various dairy products enriched with calcium, such as milk-based protein matrixes [37], dairy preparations [38], and postbiotic systems [39] have been studied for their bioavailability and efficacy. However, finding food sources rich in calcium is challenging in Asian populations due to the high incidence of lactose intolerance and/or low preference for dairy products. Therefore, chicken eggshell power, an inexpensive calcium source consisting of more than 90% by weight of calcium carbonate [21], was utilized as a calcium source for this study. In addition, elderly-friendly home meal replacements (HMRs) in the ready-to-heat category [24] were chosen for this study diet, including curry, black bean sauce, and sweet pumpkin porridge considering the age, acceptability, and convenience of study participants. All study diets were based on 100% intake, and changes in serum concentrations of carotenoids and fat-soluble pigments abundant in plants [17] indicated good compliance with the dietary intervention by study participants. Serum lutein and β-carotene concentrations significantly increased in both control and treatment groups, which continued to consume lutein-rich sweet pumpkin porridge during the intervention period. On the other hand, serum lycopene concentration significantly increased only in the treatment group that consumed tomato-added curry.

The average content of calcium and vitamin D in the study diets was 609.4 mg and 24.1 μg, respectively. Considering that study participants consumed the study diet three times a week during the intervention period, the study participants ultimately consumed an additional 261 mg of calcium and 10.3 μg of vitamin D per day through the study diet. As previously reported [40], our study participants also had insufficient calcium and vitamin D intake. However, by consuming the study diet, the total calcium intake of study participants in the treatment group was 931 mg/d and vitamin D 13.5 μg/d, reaching the recommended calcium intake of 800 mg/d [41] and the adequate vitamin D intake of 10 μg/d for Koreans. It should not be overlooked that the Korean Society for Bone and Mineral Research recommends consuming 800~1000 mg of calcium and 20 μg of vitamin D per day, and taking supplements if necessary [40]. 

However, beneficial effects of dietary calcium intake [42] or supplement use [43] on bone loss have not shown consistent results in other studies [44]. One possible explanation for these inconsistent results on the effect of calcium intake on bone loss may be related to baseline calcium and vitamin D nutritional status. Despite the small number of subjects, our study showed that bone loss can be prevented through calcium and vitamin D fortification in small amounts that reach the recommended intake in the daily diet. Given the low dietary calcium and vitamin D intake of our study participants, calcium absorption may have been efficient in our study participants, as it has previously been reported that calcium absorption is related to vitamin D concentration when blood vitamin D concentrations are low [45]. The association between supplementation and the risk of fractures can also be interpreted in the same context. A systemic review indicated that calcium or vitamin D supplementation was not associated with a low risk of fractures among community-dwelling older adults [46], and the US preventive services task force concluded there was insufficient evidence for the benefits of supplementation with more than 1000 mg of calcium and more than 400 IU of vitamin D for community-dwelling postmenopausal women [6]. It is important to note that prospective studies conducted in Japan [13,47] and China [3], where calcium deficiency is common, reported an association between dietary calcium intake and fractures in women. Consistent with the positive findings on calcium and vitamin D for bone health, our study demonstrates the beneficial effects of consuming a daily diet fortified with natural calcium and vitamin D. In addition, this study showed that eggshells, a sustainable and inexpensive source of calcium, may be a good alternative for populations who have difficulty consuming milk. 

It is important to recognize that dietary habits as well as key nutrients such as calcium and vitamin D have a significant impact on bone health, as shown in a longitudinal cohort study showing a lower incidence of hip fractures with a Mediterranean diet and adequate dietary calcium intake [48]. In addition, Asian diets rich in soy isoflavones, such as genistein, can have a protective effect against bone loss by regulating bone turnover, as reported by Marini et al. [49]. Along the same line, it should not be overlooked that tomatoes or their main component, lycopene, may exert beneficial effects on bone health in our study, given that tomatoes were added only to the study diet of the treatment group and that lycopene has been proven to prevent bone loss in an animal model [19]. 

This study was limited by the relatively small number of study subjects, and the dropout rate reached 10.7%, resulting in an actual power value of 0.615. Despite the limitation, this study demonstrated that a rapid decline in femoral neck BMD in postmenopausal women can be prevented by adding appropriate amounts of eggshell powder and vitamin D to the daily diet. Additionally, this study had limitations in that it could not quantitatively measure the amount of exercise of the subjects. It is well known that sarcopenia prevention [50] and educational interventions [51] including exercise have beneficial effects on bone health. We are currently conducting another intervention study to find answers about the effects of diet and exercise. Considering that this study was a long-term dietary intervention targeting postmenopausal women who do not prefer milk and dairy products, three types of elderly-friendly home meal plans (HMR) were used as study diets, which were well tolerated by all subjects and no adverse events were found. In the future, large-scale intervention trials are needed to evaluate the effects of different amounts of eggshell powder and vitamin D-containing foods on bone health in different age groups. Additionally, evaluating the complementary effects of tomatoes on bone health would also provide valuable information.

## 5. Conclusions

Our study demonstrated that consumption of eggshell powder and vitamin D-fortified home meal replacement (HMR) three times a week for 6 months to 1 year effectively prevents a rapid decline in femoral neck bone mineral density in postmenopausal women with calcium and vitamin D deficiency. Therefore, the current study indicates that daily consumption of a diet containing adequate amounts of eggshell powder and vitamin D may prevent bone loss and ultimately prevent fractures in populations vulnerable to calcium and vitamin D deficiency and who do not routinely consume milk and dairy products.

## Figures and Tables

**Table 1:** Baseline characteristics of the study participants.

Variables	Total (*n* = 25)	Treatment (*n* = 13)	Control (*n* = 12)	*p*-Value
**General characteristics**				
Age (years)	55.7 (3.4)	56.0 (2.7)	55.3 (4.1)	0.611
Body weight (kg)	60.1 (8.1)	59.8 (8.9)	60.8 (7.5)	0.852
Height (cm)	158.0 (4.8)	158.3 (4.4)	157.6 (5.3)	0.574
BMI (kg/m^2^)	24.1 (3.1)	23.8 (3.0)	24.5 (3.4)	0.810
Waist circumference (cm)	82.7 (7.7)	82.8 (8.0)	82.5 (7.8)	0.979
**Clinical characteristics**				
SBP (mmHg)	120.6 (10.2)	119.9 (10.7)	121.3 (10.0)	0.810
DBP (mmHg)	76.1 (9.6)	76.6 (8.7)	75.5 (10.6)	0.769
FBS (mg/dl)	96.0 (11.7)	93.7 (11.0)	98.6 (12.5)	0.270
Total cholesterol (mg/dl)	241.2 (38.2)	236.0 (32.9)	190.5 (28.9)	0.002
Triglyceride (mg/dl)	113.8 (51.4)	108.2 (51.3)	120.0 (53.1)	0.728
HDL (mg/dl)	63.9 (16.9)	68.5 (18.9)	58.8 (13.5)	0.137
LDL (mg/dl)	127.5 (35.8)	145.8 (36.3)	107.8 (23.5)	0.010
**Dietary intake**				
Calorie intake (kcal/day)	2165.7 (716.7)	2376.2 (748.8)	1972.7 (657.7)	0.082
Carbohydrate (g/d)	357.1 (122.5)	402.6 (117.0)	315.4 (116.6)	>0.999
Protein (g/d)	77.1 (27.4)	86.8 (32.0)	68.1 (19.7)	>0.999
Fat (g/d)	47.6 (18.9)	47.7 (22.0)	47.5 (16.4)	>0.999
Calcium intake (mg/d)	585.1 (24.2)	661.5 (307.0)	515.1 (142.6)	>0.999
Vitamin D intake (μg/d)	3.079 (2.5)	3.650 (2.6)	2.554 (2.4)	>0.999
**Serum calcium, vitamin D and parathyroid hormone**				
Calcium (mg/dL)	9.4 (0.4)	9.5 (0.3)	9.4 (0.5)	0.503
PTH (pg/mL)	36.0 (20.9)	41.5 (24.6)	30.0 (14.9)	0.205
25(OH)D (ng/mL)	25.1 (10.5)	25.8 (13.1)	24.2 (7.3)	0.894
**Bone turnover markers**				
Serum osteocalcin (ng/mL)	24.2 (8.2)	22.6 (7.4)	26.0 (8.7)	0.247
Urine NTx (mMBCE/mM Cr)	55.1 (24.1)	53.7 (26.8)	56.7 (21.9)	0.538
Bone Mineral Density (BMD)				
Lumbar BMD (g/cm^2^)	1.086 (0.152)	1.077 (0.148)	1.096 (0.163)	0.728
Femur neck BMD (g/cm^2^)	0.859 (0.142)	0.838 (0.117)	0.881 (0.168)	0.611
Total hip BMD (g/cm^2^)	0.930 (0.135)	0.910 (0.104)	0.951 (0.165)	0.810
**Lifestyle**				
Exercise/day (min)	60.9 (6.8)	65.8 (11.8)	55.0 (5.0)	0.872
Smoking, Yes (*n*)	25	0	0	
Alcohol drinking, Yes (*n*)	8	4	4	0.072 ^#^
Regular exercise, Yes (*n*)	22	12	10	<0.001 ^#^

BMI, body mass index; SBP, systolic blood pressure; DBP, diastolic blood pressure; FBS, fasting blood sugar; HDL, high-density lipoprotein; LDL, low-density lipoprotein; PTH, parathyroid hormone; NTx, *N*-telopeptide; BMD, bone mineral density; Exercise, aerobic exercise. Values are mean (standard deviation) and *p*-values were calculated from non-parametric comparison by Mann–Whitney U test. *p*-values ^#^ were calculated from non-parametric X^2^ test.

**Table 2:** Calcium, vitamin D, and carotenoid content in study diets.

Variables	Treatment	Control
**Calcium and vitamin D content**		
**Curry (200 g/serv.)**		
Calcium (mg/serv.)	740.1 (23.4)	101.4 (18.9)
Vitamin D (µg/serv.)	16.10 (0.62)	ND
**Black bean sauce (200 g/serv.)**		
Calcium (mg/serv.)	600.9 (13.5)	130.1 (6.08)
Vitamin D (µg/serv.)	29.95 (1.80)	ND
**Sweet pumpkin porridge (285 g/serv.)**		
Calcium (mg/serv.)	580.7 (75.41)	85.5 (1.21)
Vitamin D (µg/serv.)	26.27 (0.70)	ND
**Average**		
Calcium (mg/serv.)	609.4 (33.2)	129.2 (6.54)
Vitamin D (µg/serv.)	24.10 (0.66)	ND
**Carotenoid content**		
**Curry (200 g/serv.)**		
Lycopene (mg/serv.)	6.608 (0.049)	ND
**Sweet pumpkin porridge (285 g/serv.)**		
Lutein (mg/serv.)	6.809 (0.056)	7.008 (0.044)
β-carotene (mg/serv.)	6.211 (0.032)	6.514 (0.086)

Values are mean (standard deviation); ND, not detected. Calcium, vitamin D and carotenoids in the study diet were determined in triplicate by ICP-MS, LC-MS/MS, and UPLC, respectively.

**Table 3:** Changes of bone markers and bone mineral density after 6 months of intervention.

Variables	Treatment (*n* = 13)	Control (*n* = 12)	95% CI	*p*-Value
ΔPTH (pg/mL)	−3.9 (18.4)	5.8 (7.3)	−2.534~22.016	0.093
Δ25(OH)D (ng/mL)	−1.9 (9.5)	−3.6 (6.7)	−8.736~5.051	0.574
ΔOsteocalcin (ng/mL)	1.9 (5.7)	0.5 (6.1)	−6.347~3.400	0.650
ΔNTx (mMBCE/mM Cr)	−6.8 (17.3)	−3.2 (13.9)	−9.399~16.758	0.769
ΔUrine calcium/Creatinine	0.003 (0.088)	−0.016 (0.076)	−0.087~0.049	0.769
ΔLumbar BMD (g/cm^2^)	−0.013 (0.022)	−0.024 (0.037)	−0.121~0.139	0.320
ΔFemur neck BMD (g/cm^2^)	−0.004 (0.018)	−0.023 (0.021)	−0.035~−0.002	0.035
ΔTotal hip BMD (g/cm^2^)	−0.005 (0.015)	−0.001 (0.015)	−0.062~0.155	0.406

PTH, parathyroid hormone; NTx, *N*-telopeptide; BMD, bone mineral density. Data represent mean (standard deviation) and *p*-values were calculated from non-parametric comparison by Mann–Whitney U test. The 95% CI (confidence interval) was determined using an independent *t* test for the means of the two groups.

**Table 4:** Comparisons of bone markers and bone mineral density at 12 months of intervention and changes of each parameter.

Mean Values at 12 m/Δ	Treatment (*n* = 5)	Control (*n* = 6)	*p*-Value
PTH (pg/mL)/ΔPTH	51.8 (11.9)/12.0 (19.2)	41.8 (22.1)/15.3 (14.2)	0.537/0.792
25(OH)D (ng/mL)/Δ25(OH)D	24.9 (3.6)/−6.9 (18.0)	17.8 (4.2)/−2.6 (5.4)	0.017/0.537
Osteocalcin (ng/mL)/ΔOsteocalcin	21.3 (9.1)/1.2 (7.9)	28.1 (9.3)/0.5 (7.0)	0.177/1.000
NTx (mMBCE/mM Cr)/ΔNTx	62.0 (25.2)/14.2 (19.1)	72.8 (16.8)/5.7 (16.7)	0.537/0.329
Urine calcium/Creatinine/ΔCa/Cr	0.190 (0.064)/−0.008 (0.119)	0.132 (0.039)/−0.003 (0.072)	0.247/0.931
Lumbar BMD/ΔL-BMD (g/cm^2^)	1.071 (0.099)/−0.010 (0.028)	1.120 (0.196)/−0.035 (0.032)	0.931/0.247
Femur neck BMD/ΔFemur neck BMD	0.832 (0.137)/−0.007 (0.019)	0.887 (0.186)/−0.033 (0.020)	0.792/0.052
Total hip BMD/ΔTotal hip BMD	0.908 (0.128)/0.007 (0.021)	0.982 (0.194)/−0.020 (0.028)	0.931/0.329

PTH, parathyroid hormone; NTx, *N*-telopeptide; BMD, bone mineral density. Data represent mean (standard deviation) and *p*-values were calculated from non-parametric comparison by Mann–Whitney U test.

## Data Availability

Data are contained within the article.
